# Hypercholesterolemia induced by hypothyroidism is ameliorated by taurine supplementation through the hepatic AMPK/ACC pathway

**DOI:** 10.20945/2359-4292-2026-0078

**Published:** 2026-07-22

**Authors:** Marina Souza Matos, Georgia Correa Atella, Vânia Maria Corrêa da Costa

**Affiliations:** 1 Laboratório de Fisiologia Endócrina Doris Rosenthal, Instituto de Biofísica Carlos Chagas Filho, Rio de Janeiro, RJ, Brasil; 2 Laboratório de Bioquímica de Lipídeos e Lipoproteínas, Instituto de Bioquímica Médica Leopoldo de Meis, Universidade Federal do Rio de Janeiro, Rio de Janeiro, RJ, Brasil

**Keywords:** Hypothyroidism, taurine supplementation, lipids serum levels, dyslipidaemia, AMPK, ACC

## Abstract

**Objective:**

To evaluate the effect of taurine supplementation on the lipid profile of male hypothyroid Wistar rats.

**Materials and methods:**

Adult male Wistar rats were induced to hypothyroidism by treatment with 0.03% methimazole in drinking water. After 21 days, half of the hypothyroid animals received daily taurine supplementation by gavage (520 mg/kg b.w.), while the other half received water. Control animals received taurine at the same dose or water by gavage for an additional 21 days, totaling 42 days of treatment. The groups were: Control (C), Taurine (T), Hypothyroid (H), and Hypothyroid + Taurine (H+T) (n = 5-20/group). Data were expressed as mean ± SEM, and statistical analysis was performed using two-way ANOVA followed by Tukey’s post-test.

**Results:**

Hypothyroidism increased serum total cholesterol (TC) and LDL levels, which were reduced by taurine supplementation. Hepatic diacylglycerol concentration increased with taurine supplementation, but this effect was not observed in hypothyroid animals. Taurine increased AMPK and ACC phosphorylation in the liver independently of thyroid hormone levels.

**Conclusion:**

Hypothyroidism induces changes in the lipid profile, particularly increasing TC and LDL cholesterol levels. Taurine supplementation in hypothyroid rats partially reversed lipid alterations and increased AMPK and ACC phosphorylation in the liver, suggesting metabolic benefits.

## INTRODUCTION

Hypothyroidism is one of the most common endocrine disorders worldwide and is usually characterised by decreased production of thyroid hormones (TH) by the thyroid gland (^1^). This dysfunction is associated with a high prevalence of cardiovascular diseases and features related to metabolic syndrome, such as hypertension and dyslipidaemia (^2^,^3^). Alterations in lipid metabolism due to hypothyroidism confer a pro-atherogenic profile, with patients typically exhibiting elevated serum levels of total cholesterol (TC) and low-density lipoprotein (LDL), resulting in hypercholesterolemia. TH are known to stimulate lipolysis and the transport of fatty acids (FA) to the liver (^3^). Additionally, TH links autophagy to mitochondrial fat oxidation, promoting ketogenesis, and stimulates reverse cholesterol transport in the liver (^4^). However, TH deficiency reduces hepatic triglycerides (TG) content derived from FA and increases lipid uptake by white adipose tissue (WAT) (^5^). Cholesterol homeostasis is also regulated by TH, which stimulate its hepatic biosynthesis, uptake from circulation and conversion into bile acids (^6^). The cholesterol-lowering effect of TH can be explained by multiple mechanisms. Triiodothyronine (T3) is known to induce hepatic LDL receptor (LDL-R) gene and protein expression (^7^) and to stimulate cholesterol 7α-hydroxylase (Cyp7A1), the key enzyme in bile acid synthesis from cholesterol (^8^). Therefore, hypothyroidism is highly associated with non-alcoholic fatty liver disease (NAFLD) and is considered a risk factor for hepatic lipid accumulation (^9^).

Taurine (β-aminoethanesulfonic acid) is considered a semi-essential amino acid that is not incorporated into proteins and can be found at different concentrations in mammalian tissues. It is widely distributed in skeletal muscle, heart and brain, and its levels may vary in other tissues, such as the liver, depending on dietary intake (^10^,^11^). Many studies have shown potential benefits of taurine supplementation in cardiovascular diseases (^12^), as well as improvements in the lipid profile in obesity, NAFLD and diabetes mellitus (^13^-^15^). Taurine has been shown to stimulate FA oxidation via increased peroxisome proliferator-activated receptor γ (PPARγ) protein expression; taurine deficiency, in contrast, leads to impaired metabolism (^16^). In the same context, a taurine-rich diet was able to reduce the hepatic TG content and increase free FA levels in the liver of high-fat diet-fed mice (^17^). Furthermore, in a rodent model of obesity induced by monosodium glutamate, taurine supplementation reduced hepatic TG accumulation and serum lipid levels by modulating genes involved in lipolysis (^15^). Taurine supplementation is also associated with reduced hepatic and serum cholesterol levels, which some authors attribute to decreased HGM-CoA reductase activity, thereby reducing cholesterol synthesis (^18^) and promoting bile acid conjugation (^16^). Recent studies have identified taurine deficiency as a hallmark of aging in mice, monkeys and humans. In this context, a reversal of this decline by taurine supplementation may increase healthy lifespan across species (^19^).

Previous studies have associated lipid alterations observed in hypothyroid patients with the development of atherosclerotic disease (^20^). Even with levothyroxine treatment, some individuals with hypothyroidism show a delayed reduction in serum lipid levels, making its efficacy in improving the lipid profile by normalizing TH levels alone controversial (^21^). Increasing evidence suggests that taurine supplementation may decrease the risk of atherosclerosis by reducing oxidative stress (^22^). Therefore, this study aims to investigate the effect of taurine supplementation in the lipid profile of hypothyroid rats. We found that taurine can restore hypothyroidism-induced hypercholesterolemia, likely via modulation of the hepatic AMPK/ACC pathway.

## MATERIALS AND METHODS

### Animals

Male Wistar rats (12 weeks of age), weighing approximately 250 g, were randomly housed in groups of five in acrylic cages (18 x 31 x 38 cm) and maintained under controlled conditions from birth: temperature of 23 ± 2 ºC, a 12 h:12 h light-dark cycle (lights on at 19:00), with water and standard chow available *ad libitum*.

### Study design

This study was approved by the Ethics Committee for Animal Use in Scientific Experimentation (CEUA-CCS; No. IBCCF 080) of the Health Sciences Centre at the Federal University of Rio de Janeiro. Animals received humane care in accordance with the International Guiding Principles for Biomedical Research Involving Animals (Council for International Organizations of Medical Sciences and the International Council for Laboratory Animal Science, Geneva, Switzerland). Some animals were induced to pharmacological hypothyroidism by oral administration of 2-mercapto-1-methylimidazole (MMI; Sigma-Aldrich, USA) at a concentration of 0.03% in drinking water for 21 days, as previously described (^23^). After hypothyroidism induction, the animals were divided into four groups. Control: animals received water and chow *ad libitum* throughout the experimental period (42 days) and daily water gavage during the last 21 days; Taurine: animals received water and chow *ad libitum* throughout the experimental period and daily taurine gavage (Sigma-Aldrich, USA) at a dose of 520 mg/kg body weight (b.w.) during the last 21 days. This dose was selected based on Allen and cols. (2016), who reported that taurine treatment alone at this dose had no significant effect on the assessed parameters in healthy rats (^24^); Hypo: animals received MMI in the drinking water throughout the experimental period and daily water gavage during the last 21 days; Hypo + Taurine: animals received MMI in the drinking water throughout the experimental period and daily taurine gavage (520 mg/kg b.w.) during the last 21 days. All animals were subjected to daily gavage with drinking water for one week before the start of taurine supplementation for adaptation. Body weight was measured weekly throughout the treatment period. Three independent experiments were performed, each including five animals per group (20 animals per experiment; 60 animals in total). Sample size was calculated using http://www.gpower.hhu.de/.

### Glucose tolerance test

The glucose tolerance test (GTT) was performed on the 40^th^ day of treatment. The experimental groups were fasted for eight hours. Blood samples were then collected by tail snip, and fasting glycemia was measured using commercially available test strips and a glucometer (Accu-Check^®^Active, Roche Diagnostics). Subsequently, a glucose solution (1.75 g/kg b.w.) was administered intraperitonially (i.p.) to each rat. Glycemia was measured again from tail blood samples at 15, 30, 60 and 120 minutes after glucose administration using the same glucometer.

### Biological samples collection

After 42 days of treatment, the animals were euthanized by decapitation without prior sedation at 09:00 (dark cycle). Blood was collected from the trunk and centrifuged at 1,200 × g for 20 minutes; serum was separated and stored at -20 °C. The liver was collected, weighed, frozen in liquid nitrogen, and stored at -70 °C for further analyses. WAT depots and brown adipose tissue (BAT) were collected and weighed. Visceral WAT was defined as the sum of retroperitoneal and mesenteric depots; epididymal WAT was collected from the gonadal region, subcutaneous WAT from the anterior subcutaneous region; and interscapular BAT was analysed.

### Serum thyroid hormones, total cholesterol, triglycerides and lipoproteins measurements

Serum levels of total T3 and T4 were determined by radioimmunoassay (RIA) according to the manufacturer’s instructions (Diagnostic Systems Laboratories Inc., TX, USA). Total cholesterol, triglycerides and HDL levels were measured using colorimetric enzymatic assays, according to the manufacturer’s instructions for microplates assays (Bioclin, Belo Horizonte, MG, Brazil). Serum VLDL levels were calculated as triglycerides ÷ 5, and LDL levels were estimated using the Friedewald Equation: total cholesterol - triglycerides ÷ 5 - HDL.

### Thin-layer chromatography and high-performance thin-layer chromatography

Liver aliquots (30 mg) were used for total lipid extraction according to a previously described protocol (^25^), with modifications. A 5 µL aliquot of the extracted lipids were applied to a silica plate (Merck Millipore) as previously described (^26^). Lipid standards were applied to the plates for identification: for thin-layer chromatography (TLC), free FA, cholesterol, esterified cholesterol, diacylglycerol and triacylglycerol; and for high-performance thin-layer chromatography (HPTLC), sphingomyelin (SM), phosphatidylcholine, phosphatidylethanolamine and phosphatidylinositol (all standards from Sigma). TLC was performed using a mobile phase composed of hexane-diethyl ether-acetic acid (60:40:1 v/v/v). HPTLC was performed using a mobile phase containing acetone (7.5 mL), methanol (6.5 mL), acetic acid (6 mL), chloroform (20 mL) and water (4 mL). After development, a charring solution (3% CuSO_4_ and 8% H_3_PO_4_) was applied to the plates, which were then heated in a hood at 200 °C until complete visualization of the spots.

### Western blot

Liver samples were homogenized in Tris-HCl buffer (0.0625 M, pH 6.8) containing 10% glycerol, 3% SDS, 1 mM PMSF, 50 mM NaF, 0.01% bromophenol blue and 5% β-mercaptoethanol. Total protein concentration was determined using a bicinchoninic acid (BCA) assay (Thermo Scientific^TM^) according to the manufacturer’s instructions for microplate assays, using a bovine serum albumin (BSA) standard curve. A total of 60 µg of protein was loaded onto 7% or 10% polyacrylamide gels and subjected to electrophoresis in a running buffer (25 mM Tris-HCl, 192 mM glycine, 0.1% SDS, pH 8.3) at 120 V. Proteins were then transferred to a polyvinylidene fluoride (PVDF) membrane overnight in a transfer buffer (25 mM Tris, 192 mM glycine, 1% SDS, 20% methanol) at 20 V. Membranes were blocked for one hour at room temperature with TBS containing 0.05% Tween 20 (TBST, pH 7.6) and 5% BSA (Sigma-Aldrich) to prevent non-specific binding. Membranes were then incubated overnight at 4 °C under agitation with primary antibodies (1:1500 anti-AMPKα [2603); 1:1500 anti-phospho-AMPKα [Thr172; 50081]; 1:1000 anti-acetyl-CoA carboxylase [3676]; 1:1500 anti-phospho-acetyl-CoA carboxylase [Ser79; 11818]; all from Cell Signaling Technology) (**Table 1**). In the following day, membranes were washed with TBST and incubated for one hour at room temperature under agitation with a peroxidase-conjugated secondary antibody (1:5000 donkey anti-rabbit IgC; Santa Cruz Biotechnology; sc-2317) (**Table 1**), followed by additional washes with TBST. Immunoblots were visualized using ImageQuant™ LAS 4000 (GE Healthcare) after being exposed to a chemiluminescent substrate (Pierce ECL Western blotting substrate, ThermoScientific).

**Table 1:** Antibody specifications for Western blots

Antibody	Dilution	Manufacturer	Catalog number	Specie and type
anti-AMPKα	1:1500	Cell Signaling	Cat#2603	Monoclonal
anti-phospho-AMPKα	1:1500	Cell Signaling	Cat#50081	Monoclonal
anti-ACC	1:1000	Cell Signaling	Cat#3676	Monoclonal
anti-phospho-ACC	1:1500	Cell Signaling	Cat#11818	Monoclonal
donkey anti-rabbit anti-IgG-HRP	1:5000	Santa Cruz Biotechnology	sc-2317	Polyclonal

AMPK: AMP-activated protein kinase; ACC: acetyl-CoA carboxylase.

### Densitometry

The densitometric analysis of bands obtained from TLC, HPTLC and Western blot experiments was performed using Image J software (National Institute of Health, USA). Results were expressed as a percentage of the control group or the protein constitutive isoform.

### Statistical analysis

Data were expressed as mean ± standard error of the mean (SEM). Statistical analyses were performed using two-way analysis of variance (ANOVA), followed by Tukey’s multiple comparison post hoc test. Statistical significance was set at p < 0.05. All analyses were conducted using Graphpad Prism 7.0^®^ (Graphpad Sofware Inc., CA, USA).

## RESULTS

### Taurine supplementation does not alter thyroid hormone levels and body weight changes induced by hypothyroidism

**Figure 1:**
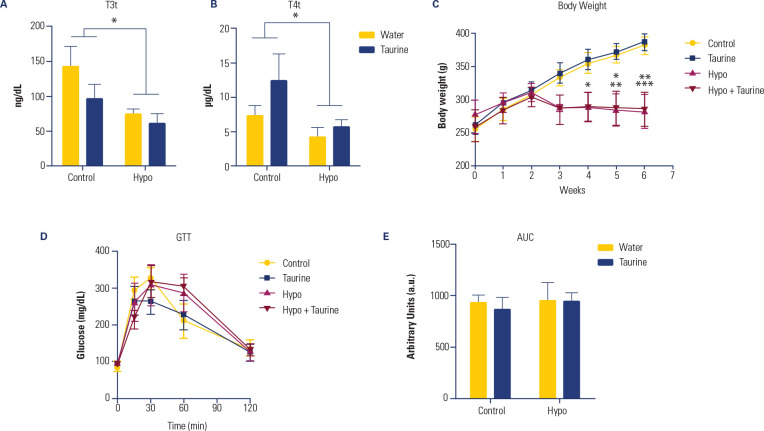
Thyroid hormones serum levels and in vivo analyses.

Serum levels of total T3 (**Figure 1A**) and total T4 (**Figure 1B**) decreased in hypothyroid animals, confirming the effectiveness of the experimental model, and were not altered by taurine supplementation. Hypothyroid rats stopped gaining weight by the end of the third week of MMI treatment (**Figure 1C**), maintaining body weight at approximately 250 g during the following weeks. Taurine supplementation was not able to prevent this alteration. In contrast, control animals, whether supplemented with taurine or not, continued to gain weight until the end of the treatment (**Figure 1C**). GTT was performed on the 40^th^ day of treatment and showed no differences in glycemia or in the area under the curve (AUC) between groups. No substantial effect of taurine supplementation was observed on the glucose profile in either group.

*p < 0.05; **p < 0.01; ***p < 0.001

### Taurine supplementation increases visceral WAT in a thyroid hormone-dependent manner

**Figure 2:**
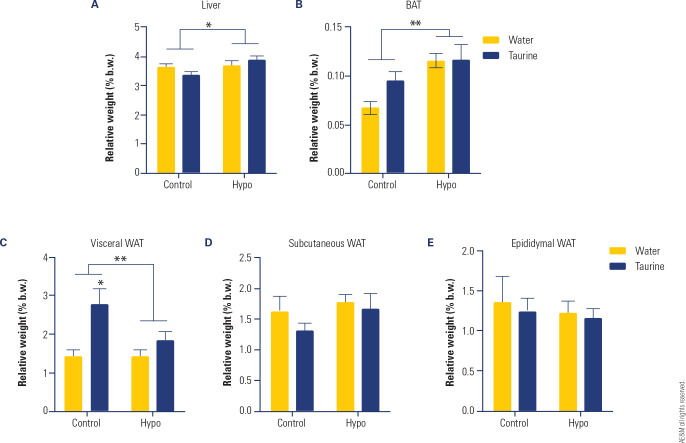
Effect of hypothyroidism and taurine supplementation in the relative weight of liver and adipose tissues depots.

The relative weights of the liver (**Figure 2A**) and BAT (**Figure 2B**) were increased in hypothyroid animals, with no influence of taurine supplementation. In a thyroid hormone-dependent manner, the relative weight of visceral WAT (vWAT) increased only in control animals after taurine supplementation (**Figure 2C**). Despite this observation, the relative weights of subcutaneous (**Figure 2D**) and epidydimal WAT did not differ between groups (**Figure 2E**).

*p < 0.05; **p < 0.01

### Hypercholesterolemia induced by hypothyroidism is reversed by taurine supplementation

**Figure 3:**
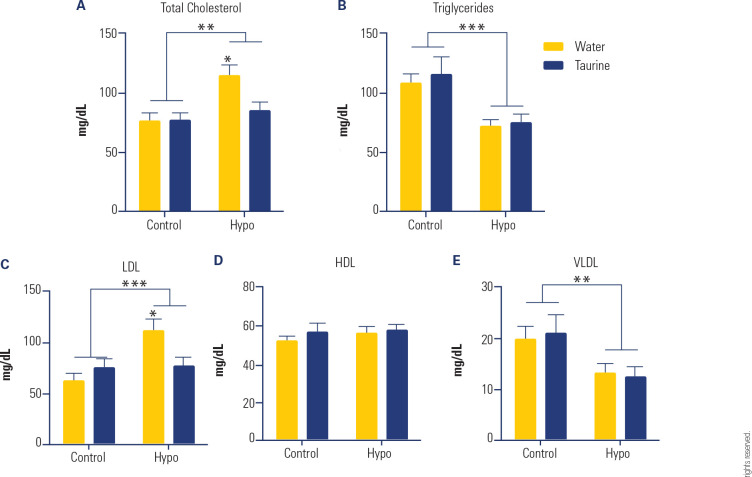
Total cholesterol, triglycerides and lipoproteins serum levels.

Serum TC levels were increased in hypothyroid animals, and taurine supplementation restored them to control values (**Figure 3A**). Similarly, serum LDL levels were increased in hypothyroid rats and were reduced following taurine supplementation (**Figure 3C**). Serum triglyceride (**Figure 3B**) and VLDL levels (**Figure 3E**) were decreased in hypothyroid animals, without alterations after taurine treatment. HDL levels were not altered by either hypothyroidism or taurine supplementation (**Figure 3D**).

*p < 0.05; **p < 0.01; ***p < 0.001

### Hepatic diacylglycerol and phosphatidylinositol contents increase with taurine supplementation in a thyroid hormone-dependent manner

**Figure 4:**
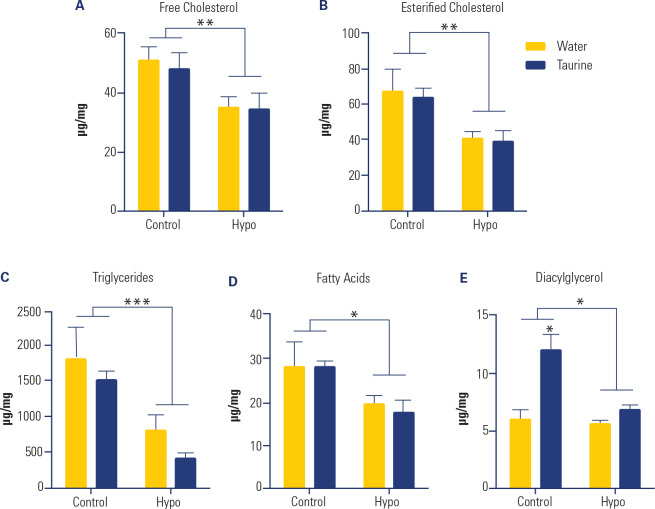
Hepatic lipid content.

**Figure 5:**
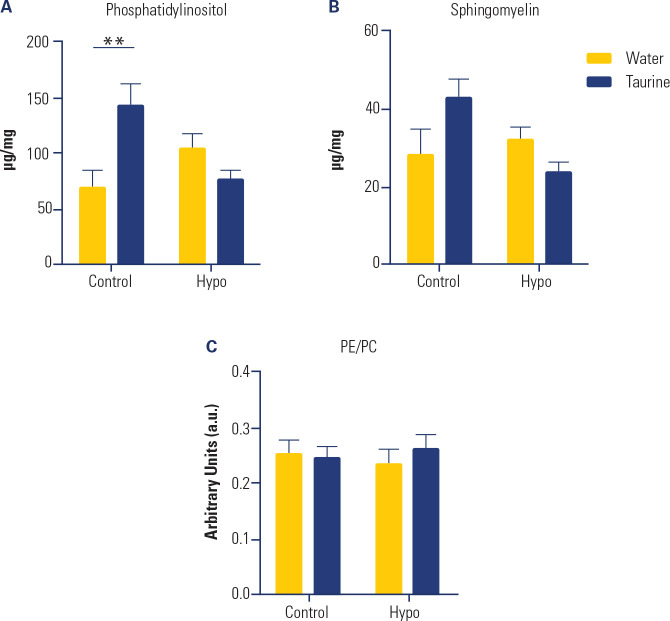
Phospholipids hepatic content.

In general, hepatic neutral lipids content - including free cholesterol, esterified cholesterol, triglycerides, FA and diacylglycerol - was decreased in hypothyroid rats, with no modulation by taurine supplementation (**Figure 4**). However, control animals supplemented with taurine showed a substantial increase in hepatic diacylglycerol levels, an effect that was not observed in hypothyroid animals, regardless of taurine supplementation (**Figure 4E**). This thyroid hormone-dependent effect of taurine was also observed in hepatic phosphatidylinositol (PI) content (**Figure 5A**). In contrast, hepatic phospholipid SM content (**Figure 5B**) and the phosphatidylethanolamine-to-phosphatidylcholine ratio (PE/PC) (**Figure 5C**) did not differ between groups.

*p < 0.05; **p < 0.01; ***p < 0.001

**p < 0.01

### Taurine supplementation increases hepatic AMPK and ACC phosphorylation independently of thyroid hormones levels

**Figure 6:**
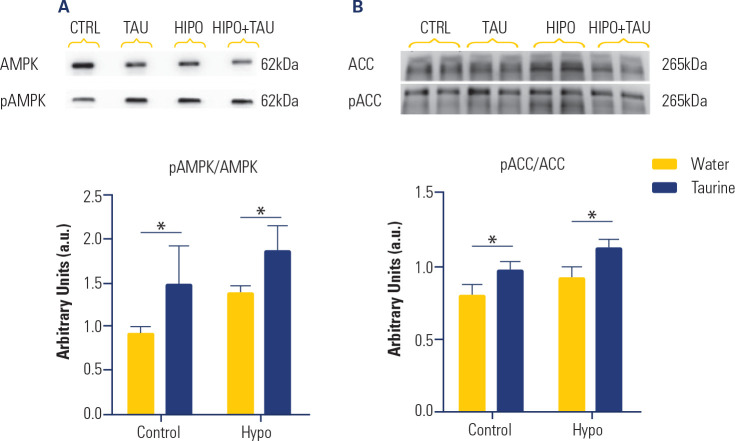
Hepatic protein levels of pAMPK and pACC.

Rats, whether hypothyroid or not, that received daily taurine gavage showed higher levels of phosphorylated AMPK (**Figure 6A**) and ACC (**Figure 6B**) compared with animals that received water only, as demonstrated by the ratio between p-AMPK/AMPK and p-ACC/ACC.

*p < 0.05

## DISCUSSION

The results presented here demonstrate that taurine supplementation during untreated hypothyroidism can reverse hypercholesterolemia, possibly by alterations in hepatic lipid metabolism.

Epidemiological data indicate that approximately 4.6% of the global population has some degree of hypothyroidism, which is commonly associated with hypercholesterolemia and elevated circulating LDL levels (^27^). Recent studies have identified regulatory factors potentially associated with dyslipidaemia in hypothyroidism, including proprotein convertase subtilisin/kexin type 9, angiogenin-like proteins and fibroblast growth factors (^28^). Dyslipidaemia associated with hypothyroidism is also frequently observed in conditions such as obesity, diabetes and metabolic syndrome (^29^). In our study, taurine supplementation did not alter body weight (**Figure 1C**) or glucose metabolism (**Figure 1D**) in either control or hypothyroid groups. However, Kim and cols. reported an anti-obesity effect mediated by inhibition of adipogenesis in animals fed a high-fat diet supplemented with taurine (^30^). Nevertheless, the role of taurine in weight gain remains controversial, with its effects depending on the animal model and study duration. In our control group fed a normal chow diet supplemented with taurine, visceral WAT mass increased (**Figure 2C**), suggesting that taurine may influence visceral adipogenesis and modulate fat distribution across different depots while maintaining overall body weight. This effect appears to be TH-dependent, as it was not observed in hypothyroid animals (**Figure 2C**). Taurine action in adipose tissue has been associated with the expression of taurine transporters in different depots. In BAT, the taurine receptor is upregulated without changes in mass in obesity models (^30^), similar to what was observed in our experimental model (**Figure 2B**).

Despite hypothyroidism being associated with insulin resistance in humans (^31^,^32^), hypothyroid rats did not show impairments in glucose homeostasis, whether supplemented with taurine or not (**Figure 1D**). Taurine supplementation has also been suggested as a potential dietary strategy for metabolic syndrome management in humans (^33^). However, our GTT-based data did not show any statistically significant differences in glucose metabolism after taurine treatment.

Regarding lipid metabolism, hypothyroidism is considered one of the major causes of secondary dyslipidaemia (^28^,^34^). Accordingly, our data show high TC levels in hypothyroid rats (**Figure 3A**), together with increased circulating LDL levels (**Figure 3C**). For the first time, we showed that taurine supplementation can decrease these lipid levels in hypothyroidism after three weeks (**Figures 3A and 3C**). Previous studies using mice fed a high-cholesterol diet showed that taurine intake for five weeks reduced TC and LDL levels, consistent with our findings (^34^). However, the exact mechanisms by which TH deficiency increases serum cholesterol levels are not fully understood. Some authors suggest that reduced LDL receptor expression during hypothyroidism contributes to elevated serum cholesterol levels. Furthermore, individuals with TH deficiency have an increased risk of atherosclerotic disease due to reduced cholesterol clearance and greater susceptibility to LDL oxidation (^35^). In fact, de Assis and cols. demonstraded that T3 supplementation modulates liver diurnal transcriptome rhythm, regulating glucose and FA metabolism and controlling hepatic energy turnover (^36^). Taurine supplementation for two weeks has been shown to increase hepatic LDL receptor gene expression in rodents fed a high-fat diet (^37^), suggesting a possible mechanism by which this amino acid reduces serum cholesterol levels in our model. Additionally, low circulating TH levels reduce cholesterol conversion into bile acids by decreasing CYP7A1 activity in reverse cholesterol transport (^4^). Moreover, a previous study showed that chronic taurine intake increased CYP7A1 activity in obese rodents with reduced enzyme activity levels (^38^). Thus, we propose that the cholesterol-lowering effect of taurine in hypothyroidism may involve some of the mechanisms described; however, further studies are necessary to confirm these hypotheses.

In line with the serum cholesterol data, hepatic levels of free and esterified cholesterol were reduced in hypothyroid rats, and taurine supplementation had no effect on their content (**Figures 4A and 4B**). These findings indicate that, under low TH levels, cholesterol efflux from peripheric tissues to the liver is reduced, resulting in decreased hepatic cholesterol accumulation and clearance. Previous studies have reported modulation of TG content after taurine supplementation in some obesity animal models (^15^,^34^), and a clinical trial in individuals with obesity associated seven weeks of taurine intake with reduced plasma TG levels (^4^). However, a transgenic mice model of obesity showed no alterations in TG content after taurine supplementation (^39^), suggesting that its effects may vary depending on the experimental model. Furthermore, taurine has been reported to inhibit diacylglycerol acyltransferase enzyme (DGAT) activity (^40^), the key enzyme responsible for the conversion of DAG into TG. This mechanism may explain the reduction in hepatic TG content observed in dyslipidaemia models supplemented with taurine (^41^). Nevertheless, further molecular studies are necessary to support this hypothesis. Our data show high hepatic DAG content after taurine supplementation in a TH-dependent manner (**Figure 4E**), which may be explained by reduced hepatic TG synthesis due to DGAT inhibition. While we did not observe changes in serum or hepatic TG levels in our experimental model (**Figures 3B and 4C**), this lack of effect may be related to the duration of taurine treatment.

Although LDL levels are increased in hypothyroidism, circulating HDL levels usually remain unchanged in this disorder (^35^), including in obesity animal models supplemented with taurine (^34^). Consistent with previous studies, serum HDL levels were also unaltered in our model (**Figure 3D**). Conversely, VLDL serum levels were significantly lower in hypothyroid animals (**Figure 3E**). This observation may be associated with low FA esterification into TG and their subsequent incorporation into VLDL (^38^), likely due to the aforementioned low TG content.

In this context, we analysed hepatic phospholipids content among groups. PI levels were higher in control animals treated with taurine compared with other groups (**Figure 5A**), suggesting elevated conversion to DAG. This finding indicates that PI may be preferentially used as a second messenger for cellular functions than being converted into TG. Furthermore, no differences were observed in hepatic SM content or in the PE/PC ratio among groups (**Figures 5B and 5C**), although SM is known to be involved in lipoprotein metabolism (^42^,^43^), and the PE/PC ratio has been reported to be modulated by intracellular taurine levels (^44^).

The effects observed may be regulated by the activation of the hepatic AMPK-ACC pathway. Our study reveals increased phosphorylation of AMPK (Thr172) and ACC (Ser79) in the liver following taurine supplementation, regardless of TH levels (**Figures 6A and 6B**). A previous taurine receptor knockout model showed reduced cardiac phospho-ACC protein expression, as well as decreased CPT1 levels (^11^). These findings support our results and suggest that taurine signalling may be essential for the phosphorylation of these proteins and, consequently, for FA oxidation and lipid metabolism homeostasis. Taurine supplementation has been recently proposed as a potential dietary strategy for the management of metabolic syndrome in humans (^33^). In fact, Attias and cols. demonstrated in rats with streptozotocin-induced type 2 diabetes that metformin combined with taurine improved metabolic parameters and protected against diabetic complications by means of antioxidative, anti-inflammatory and anti-apoptotic effects (^45^). These findings support a possible synergistic therapeutic approach involving TH and taurine to mitigate dyslipidaemia associated with hypothyroidism.

In conclusion, our results show that daily taurine supplementation is capable of improving hypercholesterolemia in hypothyroid rats. This alteration may be associated with increased phosphorylation of proteins involved in lipid metabolism, independently of TH levels. Therefore, taurine emerges as a potential candidate for the treatment of dyslipidaemia associated with hypothyroidism. However, further studies are needed to elucidate its underlying mechanism of action.

## Data Availability

datasets related to this article will be avail-able upon request to the corresponding author.
